# Absolute quantification of a plasma tRNA-derived fragment for the diagnosis and prognosis of gastric cancer

**DOI:** 10.3389/fonc.2023.1106997

**Published:** 2023-04-17

**Authors:** Shuangshuang Zhang, Yaoyao Xie, Xiuchong Yu, Jiaxin Ge, Guoliang Ye, Junming Guo

**Affiliations:** ^1^ Department of Biochemistry and Molecular Biology and Zhejiang Key Laboratory of Pathophysiology, School of Basic Medical Sciences, Health Science Center, Ningbo University, Ningbo, China; ^2^ Department of Gastroenterology, The Affiliated No. 1 Hospital, Ningbo University, Ningbo, China; ^3^ Institute of Digestive Diseases of Ningbo University, Ningbo, China

**Keywords:** tRNA-derived fragment, absolute quantification, gastric cancer, diagnostic value, prognostic value, tRF-33-P4R8YP9LON4VDP

## Abstract

**Background:**

The transition from a healthy gastric mucosa to gastric cancer is a multi-step process. Early screening can significantly improve the survival rate of gastric cancer patients. A reliable liquid biopsy for gastric cancer prediction is urgently needed and since tRNA-derived fragments (tRFs) are abundant in various body fluids, tRFs are possible new biomarkers for gastric cancer.

**Methods:**

A total of 438 plasma samples from patients with different gastric mucosal lesions as well as healthy individuals were collected. A specific reverse transcription primer, a forward primer, a reverse primer, and a TaqMan probe were designed. A standard curve was constructed and an absolute quantitation method was devised for detection of tRF-33-P4R8YP9LON4VDP in plasma samples of individuals with differing gastric mucosa lesions. Receiver operating characteristic curves were constructed to evaluate the diagnostic values of tRF-33-P4R8YP9LON4VDP for individual with differing gastric mucosa. A Kaplan–Meier curve was established to calculate the prognostic value of tRF-33-P4R8YP9LON4VDP for advanced gastric cancer patients. Finally, a multivariate Cox regression analysis was performed to assess the independent prognostic value of tRF-33-P4R8YP9LON4VDP for advanced gastric cancer patients.

**Results:**

A detection method for plasma tRF-33-P4R8YP9LON4VDP was successfully established. Levels of plasma tRF-33-P4R8YP9LON4VDP were shown to reflect a gradient change from healthy individuals to gastritis patients to early and advanced gastric cancer patients. Significant differences were found among individuals with differing gastric mucosa, with reduced levels of tRF-33-P4R8YP9LON4VDP significantly related to a poor prognosis. tRF-33-P4R8YP9LON4VDP was found to be an independent predictor of an unfavorable survival outcome.

**Conclusions:**

In this study, we developed a quantitative detection method for plasma tRF-33-P4R8YP9LON4VDP that exhibited hypersensitivity, convenience, and specificity. Detection of tRF-33-P4R8YP9LON4VDP was found to be a valuable means by which to monitor different gastric mucosa and to predict patient prognosis.

## Introduction

Gastric cancer ranks fourth in lethality among cancer-related deaths (1). A lack of obvious discomfort at the early stages of the disease is the principal reason for the low-survival rate among patients with gastric cancer. If the tumor is diagnosed and treated at a localized stage, the prognosis is improved. Using reliable liquid biopsy analysis to detect cancer-related biomarkers is convenient and non-invasive for cancer screening. Therefore, a novel biomarker that is appropriate for discriminating gastric cancer patients from healthy individuals is clinically important.

Researchers have recognized that small RNAs are crucial players in a range of biological processes (2, 3). tRNA-derived fragments (tRFs) are fragments derived from mature tRNAs or pre-tRNAs (4). Based on the cleavage site of tRNAs, tRFs can be grouped into 3’ UtRF, 5’-tRFs, 3’-tRFs, i-tRFs, 5’-tRNA halves (5’-tRHs), and 3’-tRHs (5). tRFs are involved in the pathophysiology of various diseases. For example, tRF^Val^ promotes gastric cancer progression *via* interaction with the chaperone, EEF1A1, which results in the degradation of P53 in a ubiquitin-dependent manner (2). Further, tRF3008A attenuates progression of colorectal cancer by destabilizing Forkhead Box K1 (FOXK1) in an Argonaute (AGO)-dependent manner (3).

Emerging studies have found tRFs to be abundant in various body fluids (6, 7) and dysregulated in various diseases (8–10), which suggests that tRFs may be promising biomarkers. Our team is interested in the evaluation of tRFs as biomarkers for gastric cancer. In a previous study, we found that relative to healthy individuals, gastric cancer patients had dysregulated tRF plasma profiles (11). Herein, one of these dysregulated tRFs, tRF-33-P4R8YP9LON4VDP (tRF-33), was assessed by an absolute quantification method, for its diagnostic potential as a plasma screening method for detection of patients with differing gastric mucosal lesions.

## Materials and methods

### Plasma samples

A total of 438 plasma samples were assessed, including 106 pairs of pre- and post-operative plasma samples from patients with advanced gastric cancer, 72 gastritis patients, 48 early gastric cancer patients, and 106 healthy individuals were obtained from September 2018 to October 2021. Peripheral venous blood samples were collected and centrifuged at 3000 rpm, with obtained plasma stored at -80°C before RNA extraction. Gastric mucosal lesions were identified by pathology.

The clinicopathologic data of advanced gastric cancer patients were collected. Participants with gastric cancer were not treated with antitumor agents, radiotherapy, or immuno-therapy. Moreover, patients with other malignant tumors, infectious diseases, or serious systemic diseases were excluded. All of the clinical diagnoses were confirmed by pathological examination. The study was approved by the Human Research Ethics Committee of Ningbo University (No. 2019022501). All of the participants enrolled provided signed written informed consent.

### Isolation of total RNA and reverse transcription

The total RNA from 250 or 500 µL of plasma was first extracted with TRIzol LS (Invitrogen, Germany). Then, 8 µL of enzyme-free water was added to dissolve the extracted RNA. The concentration and purity of the total RNA were determined with a NanoDrop ultraviolet spectrophotometer (Thermo Fisher Scientific, Wilmington, DE, USA). A260/A280 ratio of 1.8 – 2.1 was considered acceptable.

**Figure 1:**
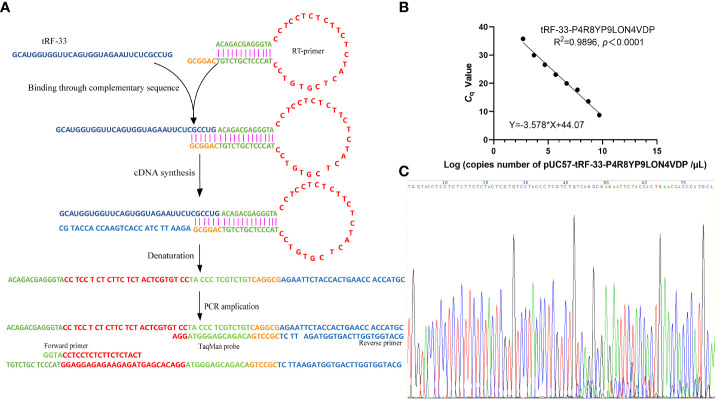
Establishment of the detection method for tRF-33-P4R8YP9LON4VDP in plasma. **(A)** Schematic representation of the method for detection of tRF-33. **(B)** The formula for the standard curve. **(C)** The T–A cloning sequencing results of the qRT-PCR product of tRF-33-P4R8YP9LON4VDP.

For the reverse transcription of tRF-33, a specific stem-loop reverse transcription (RT) primer (5’-ACAGACGAGGGTACCTCCTCTCTTCTCTACTCGTGTCCTACCCTCGTCTGTCAGGCG-3’; **Figure 1A**) and cDNA synthesis kits (Tiosbio, Beijing, China) were used. The reaction system included an RT master mix with a double-stranded specific nuclease, RT primer, total RNA solution, and enzyme-free water. The reaction temperature was 37°C for 30 min and then 85°C for 5 min.

### Quantitative PCR detection based on TaqMan probe

A quantitative real-time polymerase chain reaction (qPCR) was performed using a 5G qPCR Premix (Toroivd, Shanghai, China) containing dUTP, Mg^2+^, DNA polymerase, and PCR buffer. The reaction system contained 10 µL of the 5G qPCR Premix, 0.8 µL of the cDNA, 1.4 µL of the forward primer, 1.4 µL of the reverse primer, 0.8 µL of the TaqMan probe, and 5.6 µL of enzyme-free water. The reaction mixtures were then incubated in accordance with the instructions of the manufacturer. The primers are listed in **Figure 1A** and include: 5’-GGTACCTCCTCTCTTCTCTACT-3’ (Forward primer), 5’-GCATGGGTGGTTCAGTGGTAGA-3’ (Reverse primer), and 5’-FAM-TTCTCGCCTGACAGACGAGGGTAGGA-TAMRA-3’ (TaqMan probe).

### Construction of the standard curve for the quantification of tRF-33-P4R8YP9LON4VDP (tRF-33)

To detect the tRF-33 amount in plasma, the qRT-PCR product of tRF-33 was first inserted into plasmid pUC57 (General Biology, Anhui, China), constructing the recombinant plasmid pUC57-tRF-33. Then, eight concentration gradients, based on the initial concentration of the plasmid DNA solution (150 ng/µL) were prepared. The number of copies of the diluted plasmid DNA were calculated based on the formula: 6.02×10^23^×concentration of plasmid DNA solution (ng/µL)/(660×2783). Then, the lg (number of copies) of the eight diluted plasmid DNA concentrations were determined by transformation. The *C*_q_ values of the eight diluted plasmid DNA concentrations were determined by qPCR. According to the lg (number of copies) and the corresponding *C*_q_ values, a regression curve was constructed.

For the detection of plasma samples, the *C*_q_ value of each sample was achieved by absolute quantification. The number of plasma copies of tRF-33 were calculated based on the standard curve (**Figure 1B**) and the concentration of plasma tRF-33 (copies/mL) on the volume of total RNA.

### Statistical analysis

Data analyses were performed with GraphPad Prism v8.0 (San Diego, CA, USA) and SPSS Statistics Version 18.0 (Chicago, IL, USA). Simple linear regression was used to construct the standard curve with lg (number of copies) and the corresponding *C*_q_ values. The data of tRF-33 levels, expressed as median with interquartile, were inconsistent and fitted abnormal distribution. Tamhane′s T2 and Dunnett′s T3 were used for the analysis of inconsistent data. Diagnostic values were analyzed by constructing receiver operating characteristic (ROC) curves. A Kaplan–Meier curve was used to evaluate prognostic performance, followed by univariate and multivariate Cox proportional regression analyses.

## Results

### Detection method for tRF-33-P4R8YP9LON4VDP amount in plasma

Since tRF-33 is a short RNA of 33 nt, a hairpin RT primer was designed. The loop part of the RT primer was designed to form a discontinuous complementary sequence of 5’-ACAGACGAGGGTA-3’ and 5’-TACCCTCGTCTGT-3’ to form a stem-loop, which was connected to tRF-33 by a hydrogen bond through its 3’-sticky end (5’-CAGGCG-3’) during transcription (**Figure 1A**). Then, forward and reverse primers as well as the TaqMan probe were added (**Figure 1A**). The sequencing results of the qRT-PCR product confirmed the specificity of the primers (**Figure 1C**).

To detect the absolute amount of tRF-33 in plasma, a standard curve (Y = −3.578 × X + 44.07) was constructed based on the eight diluted plasmid DNA concentrations and the corresponding *C*_q_ values (**Figure 1B**). The correlation coefficient was 0.9896, indicating a good linear relationship for the range of plasmid DNA concentrations.

**Figure 2:**
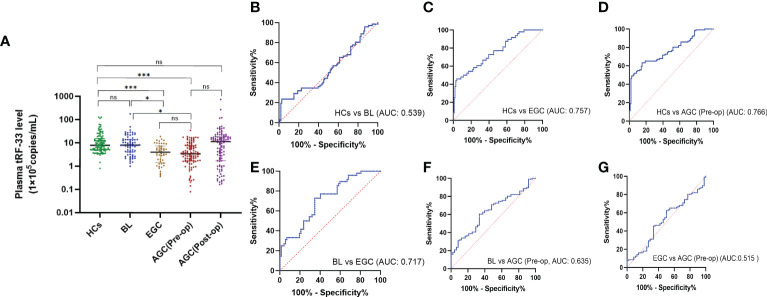
Expression levels and diagnostic values of tRF-33-P4R8YP9LON4VDP in different cohorts. **(A)** The amount of tRF-33 in the plasma showed a gradient change in healthy individuals (HC, *n* = 106), benign lesion (BL, *n* = 72), early gastric cancer (EGC, *n* = 48), pre-operative advanced gastric cancer [AGC (Pre-op), *n* = 106), and the paired post-operative plasma of advanced gastric cancer patients [AGC (Post-op), *n* = 106]. *p < 0.05, ***p < 0.001, ns, no significance. Tamhane′s T2 and Dunnett′s T3 were used. **(B)** Receiver operating characteristic (ROC) curve analysis of tRF-33 for healthy individuals and patients with benign lesions. **(C)** ROC curve analysis of tRF-33 for healthy individuals and early gastric cancer patients. **(D)** ROC curve analysis of tRF-33 for healthy individuals and advanced gastric cancer patients. **(E)** ROC curve analysis of tRF-33 for patients with benign lesion and early gastric cancer patients. **(F)** ROC curve analysis of tRF-33 for patients with benign lesion and advanced gastric cancer patients. **(G)** ROC curve analysis of tRF-33 for early and advanced gastric cancer patients. AUC, area under ROC curve.

To investigate the diagnostic significance of tRF-33 in gastric cancer, we detected the tRF-33 abundance in the plasma samples from patients and healthy individuals. The amount of tRF-33 in the plasma of patients with advanced gastric cancer was lower than that of healthy individuals (*p <* 0.001) or patients with gastritis (*p* < 0.05) (**Figure 2A**). Moreover, tRF-33 also showed lower level in early gastric cancer patients than that of healthy individuals (*p <* 0.001) or patients with gastritis (*p* < 0.05). The results suggest the potential value of tRF-33 for identification of different gastric pathologic states. However, no statistical significance was observed between the early and advanced gastric cancer patients, or healthy controls and gastritis patients. To assess the usefulness of tRF-33 for monitoring the post-operative condition of advanced gastric cancer patients, we assessed the amount of tRF-33 in paired pre-and post-operative plasma samples, however, no statistical significance was found in the paired pre-and post-operative plasma samples (**Figure 2A**). The reason might be due to small sample size and over-dispersed data distribution.

We evaluated relationships among plasma tRF-33 level and clinicopathologic parameters of advanced gastric cancer patients. First, based on cut-off value, the patients were grouped into a tRF-33 low-level group (*n* = 52) and a high-level group (*n* = 54). As shown in **Table 1**, low plasma levels of tRF-33 were related to poor tumor differentiation grade, bigger tumor size, and higher carbohydrate antigen 125 (CA125) levels in advanced gastric cancer patients. There were no relationships with other parameters such as patient age, gender, carcinoembryonic antigen (CEA), α-fetoprotein (AFP), CA199 levels, tumor-node-metastasis (TNM) stage, tumor invasion degree, or distant metastasis (**Table 1**).

**Table 1:** The correlation between the amount of tRF-33-P4R8YP9LON4VDP in plasma and clinicopathological features of gastric cancer patients.

Characteristics	*n* (%)	High (%)	Low (%)	*p*-value
All cases	106	54	52	
Gender				0.1540
Male	69 (65.09)	39 (72.22)	30 (57.69)	
Female	37 (34.91)	15 (27.78)	22 (42.31)	
Age (y)				0.1007
≥60	83 (78.30)	46 (85.19)	37 (71.15)	
<60	23 (21.70)	8 (14.81)	15 (28.85)	
CEA				0.2859
Positive	16 (21.43)	6 (11.11)	10 (19.23)	
Negative	90 (78.57)	48 (88.89)	42 (80.77)	
CA125				0.0270*
Positive	11 (10.38)	2 (3.70)	9 (17.31)	
Negative	95 (89.62)	52 (96.30)	43 (82.69)	
CA199				0.8099
Positive	21 (19.81)	10 (18.52)	11 (21.15)	
Negative	85 (80.19)	44 (81.48)	41 (78.85)	
Differentiation				0.005**
Well	17(16.04)	10 (18.52)	7 (13.46)	
Moderate	24(22.64)	19 (35.18)	5 (9.62)	
Poor	65(61.32)	25 (46.30)	40 (76.92)	
Lymph node metastasis				0.2360
N0	34 (32.08)	20 (37.04)	14 (26.92)	
N1 & N2	60 (56.60)	29 (53.70)	31 (59.62)	
N3	12 (11.32)	5 (9.26)	7 (13.46)	
Invasion				0.3990
T2	39 (36.79)	23 (42.59)	16 (30.77)	
T3	19 (17.92)	8 (14.81)	11 (21.15)	
T4	48 (45.28)	23 (42.59)	25 (48.08)	
Tumor size (cm)				0.0399*
≥5	71(66.98)	31 (57.41)	40 (76.92)	
<5	35(33.02)	23 (42.59)	12 (23.08)	
Distal metastasis				0.7585
Yes	11 (10.38)	5 (9.26)	6 (11.54)	
No	95 (89.62)	49 (90.74)	46 (88.46)	
TNM stage				0.068
I	21 (25.47)	15 (2.78)	6 (11.54)	
II	36 (28.30)	17 (31.48)	19 (36.54)	
III	40 (46.23)	19 (35.19)	21 (40.38)	
IV	9 (8.49)	3 (5.56)	6 (11.54)	
Vessel invasion				0.4411
Yes	47 (44.34)	26 (48.15)	21 (40.38)	
No	59 (55.66)	28 (51.85)	31 (59.62)	
Lymphatic invasion				0.3367
Yes	58 (54.72)	27 (50.00)	31 (59.62)	
No	48 (45.28)	27 (50.00)	21 (40.38)	
Nerve invasion				0.4449
Yes	55 (51.89)	26 (48.15)	29 (55.77)	
No	51 (48.11)	28 (51.85)	23 (44.23)	

CEA, carcinoembryonic antigen; AFP, α-fetoprotein; CA, carbohydrate antigen; TNM, tumor-node-metastasis. *: *P* < 0.05. **: *P* < 0.01.

### Clinical diagnostic significance of tRF-33-P4R8YP9LON4VDP

To assess the diagnostic monitoring potential of tRF-33 for different gastric pathologic states, we plotted receiver operating characteristic (ROC) curves for different groups of patients. As shown in (**Figure 2**) and **Table 2**, the area under the ROC curve (AUC) was 0.757 (*p* < 0.0001), differentiating between early gastric cancer patients and healthy individuals (**Figure 2C**). At the cut-off value of 334748, tRF-33 demonstrated 45.8% sensitivity, 96.2% specificity, 92.4% positive predictive value (PPV), and a 64.0% negative predictive value (NPV) (**Table 2**). We also found that tRF-33 was effective in distinguishing advanced gastric cancer patients from healthy individuals (AUC: 0.766, *p* < 0.0001) (**Figure 2D**; **Table 2**). Further, tRF-33 also differentiated early or advanced gastric cancer patients from gastritis patients (AUC: 0.717, 0.635, respectively; **Figures 2E, F**; **Table 2**). However, tRF-33 did not significantly differentiate advanced gastric cancer patients from early gastric cancer patients, or gastritis patients from healthy controls (**Figures 2B, G**). These results demonstrate tRF-33 to be efficient at differentiation of gastric cancer patients from healthy individuals and gastritis patients.

**Table 2:** The diagnostic value of tRF-33-P4R8YP9LON4VDP in monitoring gastric mucosa of different pathological states.

Groups	AUC	95% CI	SEN (%)	SPE (%)	Cut-off (Copies/mL)	PPV (%)	NPV (%)
**HC *vs* BL**	0.539	0.450-0.628	23.6	97.2	324287	89.4	56.0
**HC *vs* EGC**	0.757	0.674-0.841	45.8	96.2	334748	92.4	64.0
**HC *vs* AGC**	0.766	0.702-0.830	63.2	84.9	443348	80.7	69.8
**BL *vs* EGC**	0.717	0.626-0.808	72.9	65.3	617587	67.7	70.7
**BL *vs* AGC**	0.635	0.545-0.726	60.4	66.7	404011	64.4	62.7
**EGC *vs* AGC**	0.515	0.417-0.612	62.3	50.0	429600	55.5	57.0

HC, healthy control; BL, benign lesion; EGC, early gastric cancer; AGC, advanced gastric cancer; AUC, area under receiver operating characteristic curve; CI: confidence interval; SEN, sensitivity; SPE, specificity; PPV, positive predictive value; NPV, negative predictive value.

### Relationship of reduced levels of tRF-33-P4R8YP9LON4VDP with an unfavorable prognosis

**Figure 3:**
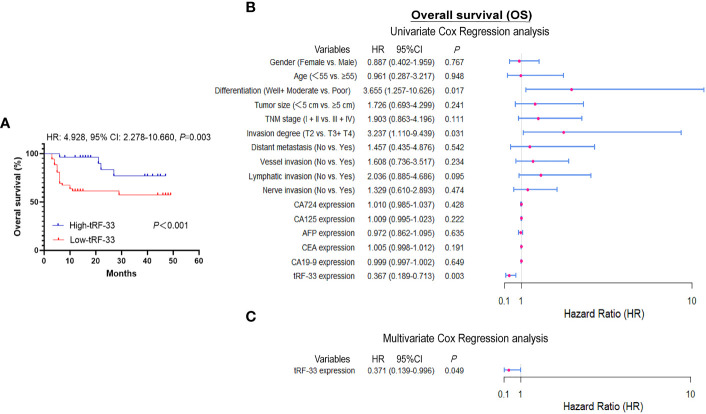
Diagnostic values of tRF-33-P4R8YP9LON4VDP. **(A)** Kaplan–Meier survival curve based on tRF-33 levels and overall survival (OS) of advanced gastric cancer (AGC) patients. **(B, C)** Forest plots of the univariate and multivariate Cox proportional regression analysis for OS in the AGC cohort.

Data for survival and follow-up were available for most of the study participants, however two patients who were lost to follow-up. Patient death was used as the clinical endpoint event for calculation of overall survival (OS), with an average approximating 21 months. By comparison of Kaplan–Meier curves, patients with reduced levels of tRF-33 had poorer survival expectancy than those with higher tRF-33 levels (*p*<0.001; **Figure 3A**). Univariate Cox regression analysis demonstrated a relationship between plasma tRF-33 level and poor survival (HR: 0.367, 95% CI: 0.189–0.713, *p*< 0.01; **Figure 3B**). Moreover, multivariate Cox analysis demonstrated tRF-33 down-regulation to predict an unfavorable prognosis for advanced gastric cancer patients (HR: 0.371, 95% CI: 0.139–0.996; *p*<0.05; **Figure 3C**), independent of other tumor markers such as; differentiation degree, tumor size, TNM stage, tumor invasion degree, or distant metastasis.

## Discussion

More than 76 million gastric cancer-related deaths have been reported and more than one million newly diagnosed gastric cancer have been reported on a yearly basis (1). According to recent GLOBOCAN statistics, the occurrence of gastric cancer varies widely in different regions. East Asians have the highest incidence, with 32.5 new cases per 10 million male residents and 13.2 new cases per 10 million female residents (1). Because of the silent symptoms of early gastric cancer, most patients are identified at an advanced stage (12). Identification of asymptomatic individuals suffering from gastric cancer is dependent upon the method of screening. Upper gastrointestinal endoscopy followed by pathologic assessment is the gold standard for gastric cancer discrimination. However, the risks for hemorrhage and perforation make this approach less desirable. With poor sensitivity and specificity for the commonly used tumor markers for gastric cancer prediction (13), there is an urgent need to develop novel gastric cancer biomarkers that improve early gastric cancer detection.

tRFs were first discovered in the1970s in the urine of cancer patients (14). In recent decades, tRFs have been identified as functional non-coding RNAs (ncRNAs) (4). Many studies have demonstrated tRFs to be ubiquitous in all domains of organisms (15–17) and to participate in various biological processes by interaction with diverse proteins or RNAs, regulating gene expression (2, 18). Moreover, accumulating evidence has shown differential tRF profiles in tumor tissues and body fluids from cancer patients (3, 19–21). Furthermore, several studies have shown that some tRFs are tissue-specific (22, 23). For example, Torres et al. demonstrated tRNA^ArgCCT^ derived 5’-tRF^ArgCCT^ to be specifically detected in the brain but not in other tissue such as testis, ovary, heart, skeletal muscle, or liver. In contrast, tRNA^GlnCTG^ derived 3’-tRF ^GlnCTG^ was found in heart, skeletal muscle, and liver but not in the brain (23). These results suggest the possibility that tRFs may be markers or molecular targets for specific cancers. Abnormal tRF expression has been associated with several diseases including cancers (24), autoimmune diseases (21), and neurodegenerative diseases (8).

Recent studies have found expression of diverse tRFs in different biological samples. For example, Xue et al. detected serum tRFs by the SYBR Green RT-PCR method (25). Wang et al. detected plasma tRF levels by relative quantification (26). In order to assess plasma tRF levels in reduced volumes, we combined the methods of specific reverse transcription and absolute quantification (**Figure 1**). This combination has been rarely reported. To detect tRF-33 in the low-volume plasma, we designed a specific hairpin structure RT primer, which effectively extended the first strand during cDNA synthesis (**Figure 1A**). The advantages of this method are as follows. First, the connection between RT primer 3’ sticky end (5’-CAGGC-3’) and the end of tRF-33 had complete sequence complementarity, which improved the efficiency and specificity of the reverse transcription (**Figure 1A**). Second, the hairpin structure of the RT primer that extended the first strand during the cDNA synthesis was beneficial for the design of amplification primers.

As there is no universally acceptable control for plasma RNA quantitation (27), we constructed a standard curve (Y = −3.578 × X + 44.07) for the purpose of absolute quantification (**Figure 1B**), which effectively improved the detection efficiency and reduced detection costs. During the amplification process, we designed a specific probe complementary to the first strand to increase the binding efficiency and subsequent amplification (**Figure 1A**). The developed detection method allowed us to achieve accurate and reliable quantification of tRF-33 in low-volume plasma.

As the development of gastric cancer is a gradual process, we were curious about whether tRF-33 was associated with dynamic changes in the gastric mucosa. We analyzed the tRF-33 levels in the plasma samples of individuals with differing gastric mucosa. Strikingly, tRF-33 showed a gradient change from healthy individuals to patients with gastritis, early gastric cancer, and advanced gastric cancer (**Figure 2A**), indicating the potential of tRF-33 for dynamic monitoring of the progression of different gastric mucosal lesions. Further, based on patient clinicopathologic data, we found that reduced tRF-33 was related to poor tumor differentiation, bigger tumor size, and a higher level of CA125 (**Table 1**), which prompted us to evaluate its diagnostic and prognostic value in gastric cancer.

Recent studies have confirmed the value of tRFs as biomarkers for the detection of various types of cancers. For example, Xue et al. identified the clinical significance of tRF^MetCAT^ and tRF^ValTAC^ in pancreatic ductal adenocarcinoma (PDAC) diagnosis with AUCs of 0.687 and 0.793, respectively (25). Wang et al. demonstrated the diagnostic values of tRF^ArgCCT-017^, tRF^GlyCCC-001^, and tiRNA^PheGAA-003^ in breast cancer by construction of joint ROC curves (26). In this study, the clinical significance of tRF-33 for monitoring different gastric mucosal lesions was assessed by ROC curves between different cohorts. In this manner, tRF-33 levels distinguished early gastric cancer patients from the healthy individuals, with sensitivity and specificity of 45.8% and 96.2%, respectively. Advanced gastric cancer patients were distinguished from the healthy individuals, with sensitivity and specificity of 63.2% and 84.9%, respectively (**Figure 2** and **Table 2**). Further, tRF-33 also showed clinical value for distinguishing early gastric cancer patients and advanced gastric cancer from gastritis patients, with sensitivities of 72.9%, 60.4%, respectively, and specificities of 65.3%, 66.7%, respectively (**Figure 2** and **Table 2**). Moreover, survival analysis of advanced gastric cancer patients demonstrated that downregulated of tRF-33 related to an unfavorable survival (**Figure 3**).

## Conclusion

We developed a method for quantitative detection of plasma tRF-33 by use of a hairpin RT primer and absolute quantification. tRF-33 was found to be a promising biomarker for monitoring gastric cancer patient progression. Results demonstrated, by multivariate prediction models, that tRF-33 is an independent variable for prediction of unfavorable survival outcomes for patients with gastric cancer.

## Data availability statement

The raw data supporting the conclusions of this article will be made available by the authors, without undue reservation.

## Ethics statement

The studies involving human participants were reviewed and approved by Human Research Ethics Committee of Ningbo University. The patients/participants provided their written informed consent to participate in this study.

## Author contributions

JuG designed the study. SZ and JuG wrote the manuscript. SZ, YX, XY, and JiG collected clinical information. GY reviewed the pathologic diagnoses. All authors contributed to the article and approved the submitted version.
